# Secure Authentication and Credential Establishment in Narrowband IoT and 5G

**DOI:** 10.3390/s20030882

**Published:** 2020-02-07

**Authors:** Jesus Sanchez-Gomez, Dan Garcia-Carrillo, Rafael Marin-Perez, Antonio F. Skarmeta

**Affiliations:** 1 Department of Information and Communications Engineering, Faculty of Computer Science, University of Murcia, 30100 Murcia, Spain; skarmeta@um.es; 2 Odin Solutions SL, 30820 Murcia, Spain; dgarcia@odins.es (D.G.-C.); rmarin@odins.es (R.M.-P.)

**Keywords:** Internet of Things, narrowband IoT, 5G, bootstrapping, authentication, EAP

## Abstract

Security is critical in the deployment and maintenance of novel IoT and 5G networks. The process of bootstrapping is required to establish a secure data exchange between IoT devices and data-driven platforms. It entails, among other steps, authentication, authorization, and credential management. Nevertheless, there are few efforts dedicated to providing service access authentication in the area of constrained IoT devices connected to recent wireless networks such as narrowband IoT (NB-IoT) and 5G. Therefore, this paper presents the adaptation of bootstrapping protocols to be compliant with the 3GPP specifications in order to enable the 5G feature of secondary authentication for constrained IoT devices. To allow the secondary authentication and key establishment in NB-IoT and 4G/5G environments, we have adapted two Extensible Authentication Protocol (EAP) lower layers, i.e., PANATIKI and LO-CoAP-EAP. In fact, this approach presents the evaluation of both aforementioned EAP lower layers, showing the contrast between a current EAP lower layer standard, i.e., PANA, and one specifically designed with the constraints of IoT, thus providing high flexibility and scalability in the bootstrapping process in 5G networks. The proposed solution is evaluated to prove its efficiency and feasibility, being one of the first efforts to support secure service authentication and key establishment for constrained IoT devices in 5G environments.

## 1. Introduction

The Internet of Things (IoT) enables advanced applications and new business opportunities in global sectors such as Industry 4.0 and Smart Cities and Precise Agriculture, among others [1]. Industry 4.0 is expanding with IoT technologies to enable advanced applications and new business opportunities. The uncertainty towards new technologies, security, and trust is one of the main obstacles to introducing Industry 4.0 service for connected manufacturing in industrial factories. The VDMA study “Industrie 4.0 Readiness” [2] reveals that only 4 of 10 industrial factory companies in Germany have a strategy for Industry 4.0, and more than 50% are beginning to engage with the concept of a smart factory. Industrial SME companies may not have the knowledge and capacity to setup and maintain connected IoT device products in a secure and trustworthy manner. Security is the biggest risk for the deployment of IoT networks in industrial SME and large companies.

Recent IoT wireless technologies (e.g., narrowband IoT (NB-IoT) [3], LoRaWAN [4], and Sigfox [5]) such as Low-Power Wide Area Networks (LPWANs) offer long-range communications and reduced energy costs, which promotes the creation of new IoT applications and services in Industry 4.0 [6]. However, these technologies pose new cyber-security challenges [7] since IoT devices have strong limitations in computing and networking capacity.

NB-IoT is one of the technologies standardized by the 3GPP and offers a seamless integration with cellular deployments from TELCOS that give coverage and support to the technology. NB-IoT is designed to be used in wide areas (several kilometres) and provides good penetration and coverage, which makes it an interesting alternative in smart cities and other use cases.

With the introduction of the new generation of cellular technologies, known as 5G, we face the situation of having several technologies—especially in the case of LPWAN, where each technology provides their own features and mode of operation. In this sense, we understand that it is paramount to provide mechanisms that unify and homogenise certain aspects of the life cycle of the IoT device, such as bootstrapping. This process involves authentication, authorization, and key management and can occur using an infrastructure that does not belong to the IoT device owner. Furthermore, the use of mechanisms to access external services (beyond the access to the network) is also important and has to be done securely. The next generation of mobile communications with 5G is already taking this into account, providing the secondary authentication schema to secure the communication with external services [8]. In this work, we take into account these features and aim to provide a solution that can be compatible with said security schema using NB-IoT.

The process of bootstrapping is a first step, prior to an IoT device being able to securely communicate in a wireless network. This process involves authentication, authorization, and key management operations, and it is vital to control network resources and data communications. Nevertheless, traditional bootstrapping is not adapted to the features of recent wireless technologies (e.g., NB-IoT) formed by IoT devices with limitations in computing and networking capacities to implement complex security protocols. In this sense, some of the bootstrapping protocols that are proposed do not account for constrained networks [9] or multi-domain scenarios. The interested reader can follow the discussion about this topic in [10].

In 3GPP cellular communication standards, bootstrapping consists of both *primary authentication* and *secondary authentication*. Primary authentication always must be successfully performed before starting any secondary authentication. On the one hand, primary authentication is managed exclusively by the network operator—it defines the mechanisms and steps necessary for a device to connect to the serving network, i.e., the 4G or 5G Core. On the other hand, secondary authentication is related to security domains outside of the serving network, thus not necessarily managed by the same entity as the 4G/5G Core. This allows flexibility with regard to communication technology for security domain administrators.

To mitigate these challenges, this paper is focused on providing a secure and lightweight bootstrapping process for the authentication and key establishment of constrained IoT devices with a focus on scenarios of the smart industry, cities, and global sectors such as Industry 4.0. In particular, this paper provides the implementation and comparison of a secure bootstrapping solution called Low-Overhead CoAP-EAP (LO-CoAP-EAP) [10], with a protocol for carrying out Authentication for Network Access (PANA), a current standard, in a real scenario of an NB-IoT network compliant with future 5G networks. The use of LO-CoAP-EAP, in this case, is complementary to the network access in NB-IoT, fulfilling the need for the secure authentication and key establishment of IoT devices with external services in a trusted and secure manner.

In this paper, we introduce a set of contributions that aim to provide secure bootstrapping and enable secondary authentication envisioned in the next generation mobile networks (5G) using the EAP lower layer known as LO-CoAP-EAP. We have implemented the protocol in an experimental testbed using real hardware devices.

The rest of the paper is organized as follows. Section 2 provides an overview of the works related to this area. Section 3 provides the background of the technologies and protocols in this work. Section 4 gives a clear use case where this contribution can be applied. Section 5 explains the design choices and the proposed architecture and testbed. In Section 6, we provide the experimental results and evaluation, and in Section 7 we have conclusions and provide suggestions for future work.

## 2. Related Work

LPWAN technologies do not provide a standardized or common solution for securing communications, bootstrapping, or the management of the life cycle of the device and the key material. As we can see in Table 1, not every solution covers key management, and in some cases ciphering the payload of the information is optional. Among the proprietary solutions, there is little that we can do to influence their future design choices, but we can demonstrate that a comprehensive secure solution is possible.

In this sense, interest in providing a solution that supports the secure management of a great number of devices—management that is envisioned to be present in an LPWAN—has been present in Internet Drafts (I-Ds) from the Internet Engineering Task Force (IETF) [7,11], as well as in a Request for Comments (RFC)of the IETF LPWAN Working Group (WG) [12], where the use of authentication, authorization, and accounting (AAA) infrastructures is portrayed as a possible future direction to manage such large numbers of devices.

There are some early proposals in IETF I-Ds to extend the LoRaWAN Joining Procedure with AAA infrastructures [13,14], which, although focused on a specific technology, serves as a demonstration that LPWAN technologies could actually benefit from their integration with AAA.

In addition to the IETF, other organizations and alliances such as Open Mobile Alliance (OMA) in their white paper *Lightweight M2M 1.1* [15] integrate different technologies including LPWAN and consider new strategies and protocols to provide security, with the incorporation of OSCORE [16], a recent IETF standard, in their protocol stack to provide security at the application layer.

The EU has also shown a clear interest in the advancement of security-related topics, through actions such as *The Next Generation Internet* (NGI) initiative by the Digital Single Market of the European Commission (https://www.ngi.eu/opencalls/ngi_trust-open-call/). NGI TRUST requests security technologies such as bootstrapping to support the development of a human-centric Internet by developing a stronger European ecosystem of researchers, innovators, and technology providers. Bootstrapping trust at the protocol level maintains a trustworthy Internet Infrastructure.

We understand from the aforementioned initiatives that the process of achieving a secure system should go through the reliance of known, tried, and tested standards, which lay the foundation for stable and trusted systems.

As the landscape of a new generation of communication technologies evolves, for example with the development of 5G [17], we can see that there is a reliance on standards to provide security to the communications. They use an Extensible Authentication Protocol (EAP) for network access and secondary authentication [18], and AAA infrastructures. Furthermore, among the different topics of the next 3GPP Release 16 [19], there are aspects about the inclusion of industrial IoT technologies, which coincides with the aim of this paper.

In our early work, we also focused on providing an LPWAN with the necessary mechanisms to secure communications, being proponents of different mechanisms to secure LPWANs [13,14,20].

In this work, we continue that effort to confirm that providing security to LPWAN technologies is possible. In previous work, LO-CoAP-EAP was tested with a real LoRa network (a LoRaFabian network) [10]; in this work, we intend to continue the process of verifying that LO-CoAP-EAP can serve the purpose of providing a bootstrapping solution for an LPWAN. In this case, we employ the NB-IoT technology. In this sense, we implement LO-CoAP-EAP to provide the device with the necessary key material to authenticate with an external server, once the device is a trustworthy part of the network, and generate the necessary key material to establish a security association with the external server.

## 3. Background

### 3.1. PANA

PANA (Protocol for Carrying Authentication for Network Access) [21] is an EAP lower layer protocol standardized by the IETF to enable authentication for network access and key establishment between devices and a network infrastructure. Following a client-server approach, PANA is a UDP-based protocol that runs between an IoT device acting as a PANA Client (PaC) and an IoT controller acting as a PANA Agent (PAA). PANA has its own re-transmission technique for the reliable delivery of EAP messages. The main payload of PANA are IP messages that enable authentication and key establishment.

**Figure 1:**
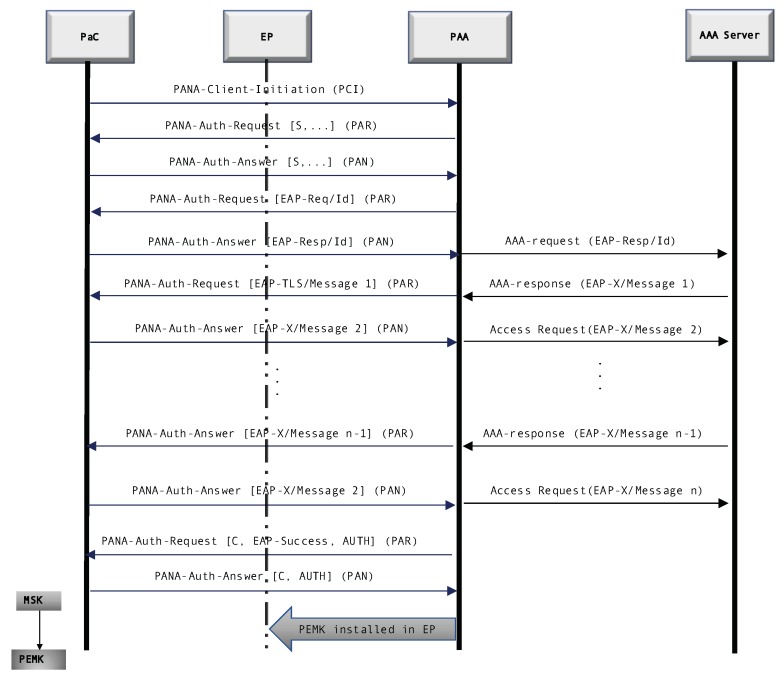
Protocol flow of PANA with a generic EAP method (EAP-X) *Source: [24]*.

For the case of PANA, we show in Figure 1, an exchange using a generic EAP method (EAP-X). At first glance, we can appreciate that it contains more message exchanges than LO-CoAP-EAP. This is because PANA was not designed with the constraints of IoT in mind. PANA uses a first message to trigger the PANA exchange (PANA Client Initiation message); afterwards, the PANA Client (PaC) and the PANA Agent (PAA) start exchanging nonces, the typical EAP request, and response identity messages, prior to the EAP method exchange. After the EAP method is finished and the PaC is authenticated, the PAA receives the EAP success message along with the MSK to derive key material and further secure communications. Details and specifics of the PANA exchange can be reviewed in work by Marin-Lopez et al. [21].

For constrained scenarios, Moreno et al. [22] developed an open-source lightweight version of a PANA client (PaC) for the Contiki Operation System (OS) [23], the so-called “PANATIKI.” PANATIKI (https://sourceforge.net/projects/panatiki/) is a PANA adaptation for wireless IoT devices with limitations in terms of computing and networking. PANATIKI follows the PANA standard, although reducing the original state machine of the PaC client to create a suitable version for limited devices. This lightweight PANA version is an optimized PANA implementation and is ideal for the comparison and evaluation of the LO-CoAP-EAP proposal for constrained devices with NB-IoT wireless technology.

### 3.2. LO-CoAP-EAP

LO-CoAP-EAP [10] is a low-overhead bootstrapping protocol integrating a Constrained Application Protocol (CoAP) as an EAP lower layer for constrained IoT devices, also known as smart objects. In fact, the LO-CoAP-EAP protocol is based on three standards: an Authentication, Authorization, and Accounting (AAA) infrastructure [25], an EAP protocol [26], and a CoAP [27]. Furthermore, this solution is included in recent standardization efforts of IETF, such as the “Secure IoT Bootstrapping” draft [28].

The LO-CoAP-EAP solution allows for a good balance of performance, flexibility, scalability, and technology independence for enabling secure authentication and the generation of fresh keying material in limited IoT devices. The integrated techniques enable an important group of features to LO-CoAP-EAP. First, the integration of the CoAP protocol allows for the implementation of a lightweight bootstrapping procedure as a service of CoAP. This solution enables a wireless technology independence because it is runs over the UDP/IP stack. Unlike PANA-based solutions, LO-CoAP-EAP is based on a CoAP, an application protocol used by most of IoT devices for machine-to-machine (M2M) communications. This reduces the code size and complexity because it does not need a new protocol to transport the EAP IP messages to perform the bootstrapping process. Moreover, LO-CoAP-EAP employs AAA infrastructures to enable enhanced properties such as identity federation, supporting more scalable deployments with multi-domain scenarios. The inclusion of an EAP allows different IoT devices and organizations to use different authentication mechanisms (based on symmetric keys, certificates, etc.) depending on their requirements. Finally, as well as PANA, LO-CoAP-EAP enables the key derivation to be employed to protect the end-to-end communications, so that the IoT device can be included as a trustworthy entity in the application domain.

The design of LO-CoAP-EAP considers the limitations of IoT devices in terms of computing capacities, low power consumption, and wireless communication technologies (LPWAN) such as NB-IoT. The following sections provide a detailed description of the developed solution evaluated in a real NB-IoT network and with 5G specifications.

**Figure 2:**
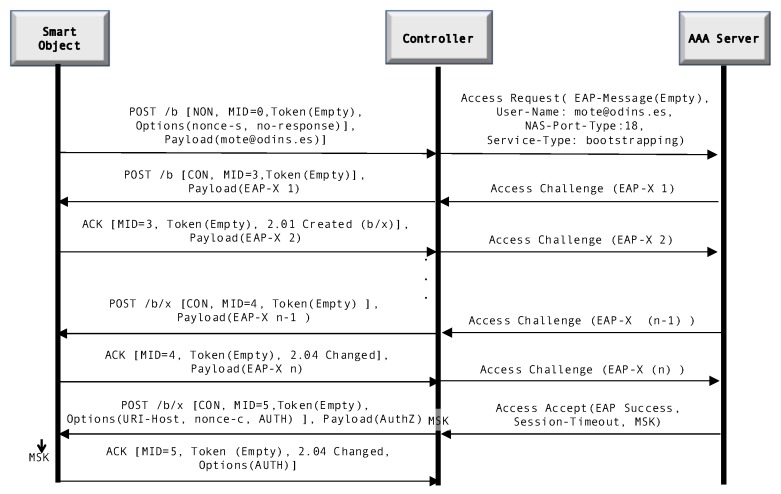
Protocol flow of LO-CoAP-EAP with a generic EAP method (EAP-X) *Source: [24]*.

Figure 2 provides an overview of the message exchange of LO-CoAP-EAP. This exchange represents the version of the protocol that does not perform a handshake. It starts with a POST message that contains the identity of the mote. The controller then creates an AAA message (RADIUS in this case), acting as an AAA client, with the information provided by the smart object and sends it to the EAP server. The EAP server then chooses the EAP method to be used for the authentication and starts the EAP exchange between the EAP server and the smart object, the controller acting as a forwarder of the EAP messages. When the EAP method finishes and the smart object is successfully authenticated, the EAP server sends an EAP success message to the controller, which contains the MSK that will be used to derive key material to further secure communications. The details and specifics of the working of LO-CoAP-EAP can be reviewed in their original work [10].

LO-CoAP-EAP represents an improvement over PANA in the realm of IoT by using CoAP. This is because CoAP is an application layer protocol that is expected to be present in most IoT stacks as a fundamental building block over IPv6/UDP [29]. It removes the necessity of application-level proxies and improves security by reducing header size, thus reducing the need of message fragmentation, which presents a security risk if an attacker can intercept fragments belonging to the same message [29]. At the time we reuse code, we are reducing the number of bytes sent over the air, as we can appreciate the simplification on the number of messages exchanged by LO-CoAP-EAP. A detailed comparison of the protocols is done in [10].

The original articles that present CoAP-EAP and LO-CoAP-EAP evaluate the protocol performance in comparison with PANA using the COOJA simulator. In this case, as we are introducing this protocol in a new technology (NB-IoT), we deliver experimental results using real devices. The experimental evaluation can be found in Section 6. Please note that NB-IoT is a Layer 2 MAC technology, as opposed to CoAP, which typically leverages UDP. As a consequence, no modifications of the NB-IoT serving network were necessary to implement our proposal. The security considerations regarding the proposed EAP lower layer in this work are inherited from the original work [10]. In this sense, we do not provide a security analysis on the authentication methods, since they have already been provided by each standard EAP method, which is in charge of providing security to the authentication exchange. The EAP lower layer needs only to be compliant with the requisites of the EAP standard [26], and, as explained in [10,30], the proposed work is indeed compliant with said requisites. Furthermore, the establishment of a security association is left to the EAP lower layer, and, in the case of LO-CoAP-EAP, we use an authentication tag implemented in a CoAP option named AUTH, which contains an HMAC of the exchanged messages between the controller and the smart object once the EAP authentication is successful, using the key material derived from the EAP Key Management Framework [31].

### 3.3. Narrowband IoT

Narrowband IoT (NB-IoT) is a new radio interface for LPWANs standardized by the 3GPP. It is one of the most promising IoT standards defined in the *3GPP Release 13*, together with Long-Term Evolution (LTE) for Machines (LTE-M), and has been extended in Releases 14 and 15. The NB-IoT design requires support for a massive number of low-throughput devices connected to the same site cell. This equates to 40 devices per household in a densely populated area such as London [32]. NB-IoT was designed as a communication technology for power constrained devices that are expected to operate during the months or years supplied by a single battery charge with a capacity of 5 Wh. In this regard, NB-IoT implements two power saving schemes, namely, Power Saving Mode (PSM) and Extended Discontinuous Reception (eDRX).

NB-IoT employs a single narrowband of 200 kHz with low baseband complexity to maintain a lower cost and a large coverage range. Technically, NB-IoT has a link budget of 164 dB, significantly better than previous standards. For instance, compared to legacy GPRS technology, NB-IoT counts with an extended coverage of 20 dB. This grants NB-IoT extended coverage ranges of up to 10 km in line-of-sight scenarios and of up to 1 km in densely populated areas. The radio modulations of NB-IoT in the Physical layer (PHY) are Binary Phase Shift Keying (BPSK) and Quadrature Phase-Shift Keying (QPSK) for the uplink and downlink, respectively. The access scheme employs Single-Carrier Frequency-Division Multiple Access (SC-FDMA) for the uplink communications and Orthogonal Frequency Division Multiplexing Access (OFDMA) for the downlink. In both cases, it employs subcarriers of 15 kHz in half-duplex communication mode.

Data rates range from 160 bps to 250 kbps, depending on scenario conditions. Most IoT applications have permissive latency requirements, with a target latency of 10 s or less. It is expected that by 2025 the number of deployed NB-IoT devices will surpass 5 billion devices. As a consequence, NB-IoT devices are targeted to be marketed with prices of US$5 per unit. NB-IoT development is based on LTE and supports all the signaling needed to establish communications with a LTE base station. Thus, any LTE compatible base station can be upgraded via software to support NB-IoT. This characteristic greatly reduces the deployment time and complexity of operations. NB-IoT can be deployed in three different operation modes, namely (i) Inband, (ii) Guardband, and (iii) Standalone. In Inband mode, the device employs a resource block of 180 kHz within a LTE carrier band. In Guardband mode, the energy is irradiated in the unused guard resource blocks between two LTE carriers. In Standalone mode, it occupies one GSM channel, and not LTE radio band resources.

## 4. The Use Case of Precise Agriculture

Regarding recent forecasts of IoT use [33], the deployment of IoT networks is expected to rise from 25 billon devices in 2018 to more than 125 billon devices in 2025. Furthermore, the global IoT market is expected to reach USD 1.567 trillion by 2025. The IoT networks are fundamental for novel services and applications in almost all markets such as precise agriculture, smart cities, and smart factories, among others.

To achieve precise agriculture, the farming sector has highly benefited from Wireless Sensor Network (WSN) technologies and is expected to equally benefit from IoT technologies [34] to address recent problems. Some problems such as water quality degradation, underground water depletion, demographic imbalances between rural and urban areas, and the soil salinization process have become evident in arid and semiarid areas [35]. Some of these problems are especially relevant in the Mediterranean region, in which large areas are currently vulnerable to water scarcity and drought events [36]. In the Mediterranean region, irrigated agriculture contributes 75% to the final production. There is a high demand for secure IoT technologies that increase water use efficiency and make additional (non-conventional) water sources available for fertigation, thereby decreasing water scarcity and the discharge of water and nutrients to the environment. These IoT technologies enable precise agriculture to optimise the sustainability of agriculture using precision irrigation techniques, adopting and implementing new water and nutrient management practices.

In particular, precise irrigation methods are rapidly developing in order to save water while improving yields and fruit quality. Although irrigation has been practised for centuries, precision irrigation is a new issue, as the sector has had to respond to societal demands for reductions in water allocation and improvements in efficiency. Irrigation strategies have been proven to successfully increase Water Use Efficiency (WUE) by reducing water use. Thus, the current trend of fertirrigation management implies (i) precision crop irrigation and fertigation, (ii) the use of crop-based information (crop indicators/descriptors), (iii) enhanced analysis, interpretation, and vaporization of the collected data, and (iv) the development of systems for growers’ aid on efficient fertirrigation control.

Recent IoT technologies formed by embedded devices (i.e., sensors, gateways, etc) and low-power wireless technologies are being deployed [37] for precise agriculture. In particular, there is a wide variety of possible IoT sensors available on the market for monitoring the conditions of plants, soils, and the environment [38]. The IoT family of innovative wireless technologies is capable of offering many solutions towards the modernization of agriculture. The IoT world offers new LPWAN networks (Low Power Wide Area) such as Sigfox, LoRa, NB-IoT, and 5G to allow wireless and autonomous devices for monitoring and control without expensive routers or repeaters. LPWAN technologies enable the Internet connection in disperse agriculture places without traditional connections based on 3G/4G cellular technologies. To integrate legacy agriculture machinery, IoT gateways are used to enable the interaction with crop facilities to perform precise data acquisition and enhanced remote actuation over nutrition pumps, irrigation valves, and greenhouse actuators (e.g., ventilation or lighting). Finally, the analysis of IoT data allows for interpreting and valuing the collected data of complex processes, predicting situations, and improving irrigation decisions.

However, the lack of standardized security solutions for IoT technologies is one of the main obstacles to deploying IoT devices with wireless communication in agricultural fields. International activities are fostering vendors and users towards IoT products with high security/trustworthiness by standardization efforts and regulations (e.g., ENISA and the EU Cybersecurity Act) [39]. Moreover, cyber-criminals have already discovered the potential of exploiting vulnerabilities of IoT devices as tools to attack industrial sectors such as agriculture. Farmers especially are vulnerable to cyber-attacks because they do not have the knowledge and capacity to setup and maintain connected IoT devices in a secure and trustworthy manner. For these reasons, security is the biggest risk of IoT deployment in the agriculture sector.

Regarding the features of precise agriculture, this use case presents an excellent environment for showing the importance of the proposed security solution for the deployment and secure communications of IoT devices with NB-IoT technology. In this environment, IoT devices are deployed to collect sensitive agriculture data that must be sent to data-driven decision support systems so that they can perform automatic actions that optimise the efficiency of critical resources (i.e., water, soil, and nutrients). In precise agriculture, the use of IoT technologies requires addressing security aspects related to the deployment and setup of the communications of constrained devices with low-power wireless technologies [40].

**Figure 3:**
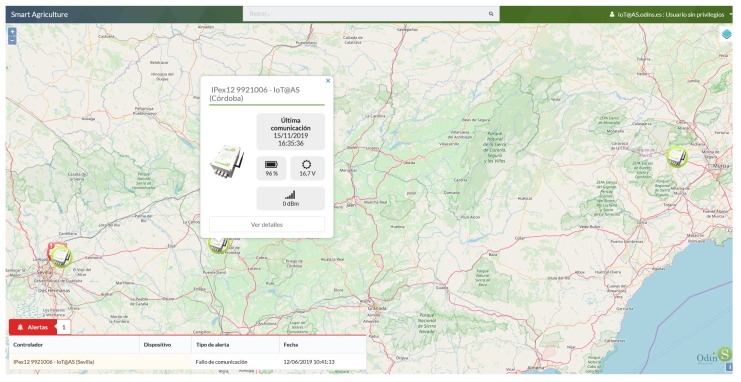
Screen-shot of a precise agriculture platform.

The proposed environment is a real pilot farm located in Santomera (Southeast of the Murcia Region, Spain). The environment is presented in Figure 3, where the locations of several deployed IoT devices are plotted. This screen-shot was captured from the data-driven agriculture platform for the precise irrigation of crops. The data-driven platform creates automatic actions regarding the gathered data from the IoT devices.

For data gathering, IoT devices were deployed to monitor several features of agricultural crops, such as water consumption, soil moisture, and weather conditions. Each IoT device acts as a data source collecting agricultural information from the field and sends the corresponding data to the smart platform employing Internet protocols (i.e., CoAP). All data from IoT devices are accessible to end users through the web interface of the smart platform located in the cloud. Nevertheless, these data may be compromised by cyber-attacks in the case that security mechanisms are not integrated.

**Figure 4:**
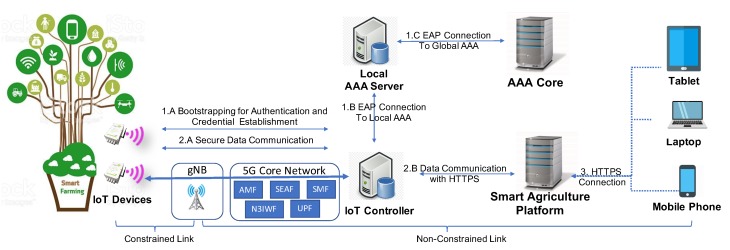
Diagram of a precise agriculture use case.

Therefore, this specific agriculture scenario is used for the demonstration of the proposed security solution integrating a group of modules to allow the secure data communication between IoT devices and the data-driven platform. To reach that, the presented use case develops our bootstrapping solution integrating the LO-CoAP-EAP protocol in NB-IoT technology for the secure authentication and credential establishment. Below, the bootstrapping procedure is described to enable the secure joining of IoT devices when they are deployed in the network and application domains. This bootstrapping generates the needed credentials between the IoT device and the IoT controller using the LO-CoAP-EAP implementation, as shown in Figure 4. In the figure, multiple IoT devices are deployed to communicate with the IoT controller through an NB-IoT base station connected to a 4G/5G core network. The IoT controller is in charge of authenticating the new IoT devices and supporting the secure data communications with the agriculture platform through protected channels. The procedure consists of the following steps: first, an IoT device must be authenticated against the serving network to start the connection with the IoT controller before sending any data to the application domain. Once the IoT device is connected to the NB-IoT base station (i.e., gNB), the IoT device begins the authentication using the bootstrapping protocol with the IoT controller through the NB-IoT gNB (Stage 1.A). In the case, the IoT device belongs to a local organization, and the IoT controller will connect the local AAA server using the RADIUS protocol (Stage 1.B), as abovementioned. Otherwise, for external organizations, the IoT controller will go to the global AAA server of the IoT device organization (Stage 1.C), as described in our previous papers [10,30]. When the bootstrapping has finished, the IoT device and the IoT controller will obtain the required credential material, as described in Section 5. After that, the IoT device can perform a secure data communication with the IoT controller, employing the credentials generated during bootstrapping. In Stage 2.A, the IoT device can use the best suited EAP lower layer to exchange data with the IoT controller in a secure and trustworthy manner. Finally, in Stage 2.B, the IoT controller without computing limitations can exchange data with the smart agriculture platform in a secure way using the HTTPS protocol integrating HTTP with Transport Layer Security (TLS) [41].

## 5. Architecture Description and Evaluation

### 5.1. The Proposed Architecture

**Figure 5:**
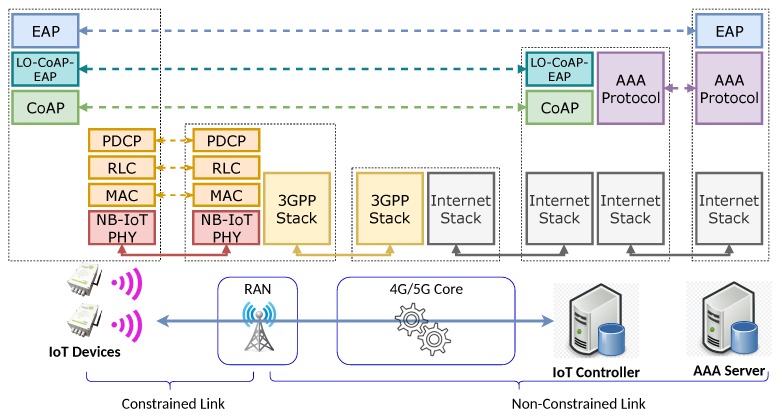
The proposed architecture for lightweight authentication and key establishment over NB-IoT.

The proposed architecture of this work is showcased in Figure 5. It addresses lightweight authentication and key establishment for NB-IoT IoT devices. It is of note that the architecture is generic for both a Radio Access Network (RAN) and an authentication protocol. In this sense, the RAN employed in its implementation could be LTE in the case of 4G or New Radio (NR) for the case of 5G systems. Likewise, for the case of the authentication protocol, the proposed architecture may be implemented for the best suited EAP lower layer. The IoT device is the device that needs to perform the authentication and key establishment process. It contains the needed hardware and software to perform a connection to the Core through an NB-IoT RAN code and include at least one NB-IoT-compatible radio modem that is capable of communicating over the chosen radio band configured by the Mobile Operator. Hence, any resource-constrained IoT device that is capable of implementing the authentication binary code will be compatible with this proposal. More details on this are presented in Section 6.1. The RAN represents the end of the constrained radio link and is composed by base stations, compatible with either LTE (eNodeB) or NR (gNB), depending on the specific implementation. The Core is the element that manages the IoT device’s serving network and its connection to data networks, in this case, the Internet. The IoT controller is the EAP authenticator that steers the process; it implements the lightweight authentication protocol employed by the end-device. In addition, the IoT controller communicates through an AAA protocol with the AAA server.

First, when powered on, the IoT device will perform the RAN attachment against a base station. Once attached to the base station, it will connect to the serving network provided by the Core. Next, in turn, the Core will activate the required mechanism that provides the functionality of sending packets to the external data network, i.e., the Internet. The IoT device will then send the user plane IP/IPv6 packets addressed to the IoT controller. These packets carry an authentication protocol data unit (PDU), which can be instantiated in either PANA or LO-CoAP-EAP. Finally, the IoT device will establish a security association with the IoT controller through the EAP. As a result of this process, the IoT device will be considered as authenticated and derive crypto key materials.

### 5.2. Performance Analysis with Real Devices

For the purpose of validating the proposal, the most relevant performance parameters of NB-IoT in recent literature were considered [42]. The requirements of IoT deployments identified are as follows: (i) an extended coverage range, (ii) device cost, (iii) efficient energy management, and (iv) scalability to serve a massive amount of devices per cell. Regarding NB-IoT energy management, 3GPP introduced two power saving schemes in Releases 13 and 14, i.e., PSM and eDRX. These modes allow the UE to enter a state where monitoring the signaling and scheduling information is not needed [43]. There is already research available on the energy performance of NB-IoT with regard to this. For instance, the authors in [44] presented empirical power consumption measurements on early, commercially available NB-IoT hardware. The results show that a device transmitting 100 bytes of payload every 24 h can operate for more than 12 years with a C-cell battery. The authors of [43] presented and validated a model of NB-IoT performance with regard to battery lifetime and latency. The results indicated that power does not grow in a linear manner in terms of packet length; instead, it is worsened due to the segmentation of longer packets. Moreover, adverse channel conditions will require retransmissions that increase power consumption. The work in [45] presented an analytical model to measure the impact of channel conditions on NB-IoT communications. The results show that, at a Maximum Coupling Loss (MCL) of 145 dB or more, the performance of the system starts to be affected with regard to battery lifetime and latency. At a 164 dB MCL, the battery lifetime is reduced by 90% compared to ideal conditions. In addition, devices that report periodically in shorter intervals will consume more energy. This is because most of the power is consumed during transmission [46]. Similarly, scalability is also one of the design goals of NB-IoT. It is aimed to serve 40 devices per household—in urban areas similar to London, this equals approximately 50,000 devices per base station [47]. In addition, the authors in [48] provided an analysis of NB-IoT performance in terms of capacity for realistic use case scenarios. They measured the performance of channel occupancy of both downlink and uplink with regard to different device transmission intervals and base station density. Their results suggest that a system is able to cope with up to 12,000 packets per hour per base station.

After evaluating all the aforementioned works, a reduced message size and a reduced number of packets required were considered as apt performance indicators for this validation process. It has reasonably been shown that they are tightly related to the energy, scalability, and latency performance of NB-IoT communication systems.

In order to validate the proposed architecture, a real-life implementation was carried out as a proof of concept. As a result, a testbed was built with real hardware while taking into consideration the constrained nature of IoT devices. Thus, the hardware was chosen with the specific purpose of replicating the characteristics of a typical scenario. An analysis of different parameters of a practical nature for the scenario are presented next.

### 5.3. Testbed

All authentication-related communications took place between two end-points, namely, the IoT controller and the IoT device. A single IoT controller handled all the authentication requests to the centralized AAA server. Both the IoT controller and the AAA server were co-located in an Intel i5-7400 CPU at 3.00 GHz with 8 GiB of system memory. Moreover, the IoT device was mainly composed of two different elements: an Arduino-compatible SmartEverything FOX board by Arrow and an NB-IoT compatible radio module. The SmartEverything board employed as an IoT device contained an ATMEL SAMD21 Ultra low-power ARM Cortex-M0+ microcontroller. The ATMEL SAMD21 had 256 kB of programmable flash storage and 32 kB of main memory. Additionally, the NB-IoT radio device employed was the Quectel BG96 Multi-mode module, configured to use LTE Cat NB1 only (NB-IoT). Due to the lack of 5G deployments in the nearby area, the employed backhaul network was a 4G system. It was composed of an NB-IoT LTE RAN and a 4G Evolved Packet Core (EPC). Without a lack of genericity, this same scenario could be achieved in a 5G system with LTE RAN or New Radio (NR) indistinctly. During all the experiments, the IoT device was static and located at approximately 575 m from the closest eNodeB.

The service network employed during the experiments was a public 4G network managed by a mobile operator company. To gain access, the IoT device included a USIM card with a limitless data subscription plan. Additionally, during the experiments, a laptop computer was employed in the field. The laptop was connected through a serial console cable to the IoT device during the duration of all the experiments. The IoT device included a special debug code that exposed a customized command interface that controls several debug functions. Using a script, the laptop triggered the bootstrapping process from scratch by communicating with the IoT device through the command interface. This command was sent to the IoT device at random times of the day. During the bootstrapping process, the laptop logged all the serial console messages of the IoT device, including time-stamps and message sizes at the time of encode and decode. Both PANATIKI and LO-CoAP-EAP were employed in the experiments. Additionally, the IoT controller and AAA server logged all the network packets using *tcpdump* during the whole process. These logs were later reviewed to match the data logged by the laptop connected to the IoT device. Finally, no notable adverse weather events took place during the duration of the process—the field equipment remained safe and was never compromised.

### 5.4. Messages Size & Overhead

**Figure 6:**
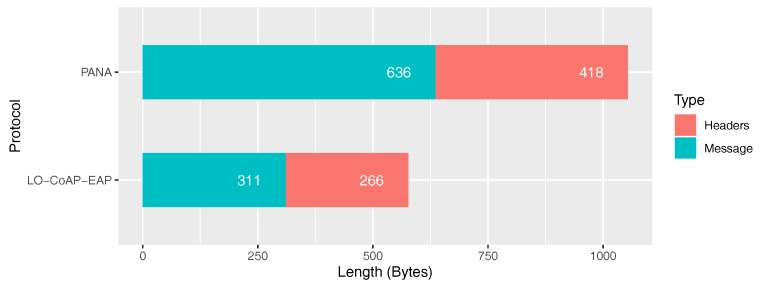
Total sum of authentication process messages and header sizes grouped by protocol.

Table 2 presents a summary of the authentication message sizes and their respective NB-IoT LTE PDU length. The application payload of NB-IoT LTE for the authentication process carries the IP packets outside of the 4G system. The figure in the *Message Length* column in Table 2 does not take into account lower layer protocol headers. On the contrary, the figure of the *NB-IoT PDU Length* column considers the headers of NB-IoT, IP, and UDP, with values of 10, 20, and 8 bytes, respectively. Furthermore, the LTE RAN of NB-IoT employs a stack consisting of three different protocols, from lower to higher positions in the stack: Medium Access Control (MAC) [49], Radio Link Control (RLC) [50], and Packet Data Convergence Protocol (PDCP) [51]. Each of these protocols has a variable length header, and its size depends on several circumstances related to the previous attachment of the UE to the eNodeB. In addition, during the attachment process, signaling data is exchanged between the UE and the eNodeB, which serves the purpose of readying both entities for the following user plane exchanges. Hence, an exact figure of the total overhead introduced by the NB-IoT LTE protocol stack cannot be achieved due to the variability of header sizes in each message exchange. Thus, for the purpose of the overhead analysis, an average header length value of 10 bytes was fixed, which conveys the entire NB-IoT LTE protocol stack. As a result, a summary of the total messages and header sizes is presented in Figure 6.

## 6. Experimental Results

### 6.1. Code Footprint

Code footprint was also taken into consideration during the developing of the testbed. For this reason, the development employed libraries targeted at constrained devices in order to maintain a low programmable memory size. For peripheral communications with the Quectel BG96 NB-IoT modem, a modified version of the *Tuino Zero 96* library (https://github.com/gimasi/TUINO_ZERO_96) was employed. Mainly, the modifications encompassed an implementation in transparent mode for the IP/UDP sockets. This library was employed in both PANA and LO-CoAP-EAP experiments. In both cases, it had a footprint of 2571 bytes of programmable memory. The libraries employed for managing the EAP authentication process were based on *PANATIKI*, which is a PANA implementation for the Contiki OS (https://github.com/contiki-os/contiki). In the case of LO-CoAP-EAP, the EAP libraries were adapted to work in the Arduino framework and encode/decode CoAP packets. Likewise, the PANA implementation of the testbed required some adaptation of the *PANATIKI* libraries in order to work with Arduino. After the adaptation process, the compiled code size was 8916 bytes in the case of LO-CoAP-EAP and 7586 bytes for PANA. In either case, the total sum of the Quectel BG96 libraries and EAP authentication libraries stayed below 11.5 KiB. Thus, this implementation was considered reasonable for a wide variety of constrained IoT devices that can manage our proposal efficiently. For instance, a similar device to the one employed in this scenario is the *Arduino Nano*, which also includes the SAMD21 MCU at 48 MHz and 32 KB of SRAM. As a consequence, the minimum hardware required to achieve authentication would be similar to that of a *Class 1* constrained device as defined in [9]—that includes any NB-IoT compatible radio modem with UDP support, e.g., *Quectel LTE BC95*.

### 6.2. Runtime

**Figure 7:**
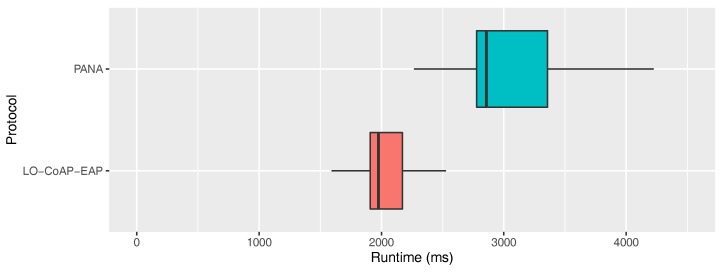
Authentication process total runtime distribution.

During the execution of the real-life experiments, the total elapsed time needed to perform a complete authentication process exchange was measured for each protocol. For each iteration of the experiment, all the packets detailed in Table 2 were exchanged for each protocol. First, a time mark with a precision of milliseconds was logged in the IoT device at the beginning of the procedure. Next, both the IoT device and the authentication server sent and received all the packets needed for the full exchange. Finally, when the IoT device received the last piece of information needed to complete the authentication process, an end time mark was logged. Note that this measurement includes all the real-life computational times of the IoT device, the intermediate routing elements, and the authentication server. Additionally, this measurement also includes any delays introduced by the real-life NB-IoT LTE network routing elements. Due to the high statistical skewness and kurtosis levels of the average results, the median was chosen instead as a better representation of this measurement. For the case of the LO-CoAP-EAP protocol, the measured value had a median of 1975 ms with a confidence interval of 1971–1982 ms over the median with a confidence level of 95%. Similarly, for the PANA protocol, the elapsed time had a median of 2857 ms with a confidence interval of 2849–2862 ms over the median, also with a confidence level of 95%. Figure 7 presents the aforementioned results.

## 7. Conclusions and Future Work

Bootstrapping is critical for the secure authentication and credential establishment in NB-IoT and 4G/5G networks. This paper has proposed the adaptation of lightweight bootstrapping protocols to enable a secondary authentication feature of 5G for constrained IoT devices. In fact, this approach represents the adaptation and evaluation of both PANA and LO-CoAP-EAP, to provide high flexibility and scalability in the bootstrapping process. We adapted two EAP lower layers, i.e., (i) PANATIKI as a IETF standardized EAP lower layer and (ii) LO-CoAP-EAP. The latter is a new EAP lower layer specifically designed for IoT and provides high flexibility and scalability in the bootstrapping process. We have compared their leveraging abilities in NB-IoT and 4G/5G environments. Moreover, this paper has explained how limited IoT devices can bootstrap and establish credentials in real scenarios with a 4G/5G network and an AAA infrastructure. Furthermore, the solution allows for deriving credential keys between a limited IoT device and an external IoT controller to create secure channels for protecting M2M data exchange. To validate the proposal, we have implemented, deployed, and evaluated a pilot testbed with real limited IoT devices as part of the proposed use case of precise agriculture. In this case, real devices are connected via NB-IoT modules to a 4G core network and an AAA infrastructure. The performance results demonstrate that LO-CoAP-EAP is a feasible and efficient solution to be used in NB-IoT and future 5G networks to enable the secondary authentication and the security association of limited IoT devices.

As future work, we consider the implementation of OSCORE to provide efficient secure end-to-end communication for constrained devices, as well as the inclusion of compression techniques to minimise the payload sizes and the number of messages required for the bootstrapping process. Furthermore, the proposed solution can be employed in other applications (i.e., smart cities and smart factories) where the efficient and secure deployment of NB-IoT devices is crucial. Although we presented payload size and runtime latency as performance indicators, further experiments with different IoT device configurations and requirements shall be considered. New results could suggest the level of fitness of the proposal to each specific use case or scenario, together with modifications that would clearly improve its current design.

## Figures and Tables

**Table 1:** High level description of the security features of several Low-Power Wide Area Network (LPWAN) technologies.

Technology\Feature	Authentication	Integrity	Cipher	Key Management
LoRaWAN	YES	YES	YES	YES
Sigfox	YES	YES	Optional	NO
Weightless	YES	YES	YES	YES
NB-IoT	YES	YES	YES	YES
DASH-7	YES	YES	YES	NO
RPMA	YES	YES	YES	YES
WiSUN	YES	YES	YES	YES

**Table 2:** Length of messages exchanged in each protocol.

Protocol	Message	Message Length	NB-IoT PDU Length
	POST	29	67
	POST(EAP-PSK1)	36	74
	ACK(EAP-PSK2)	69	107
LO-CoAP-EAP	POST(EAP-PSK3)	68	106
	ACK(EAP-PSK4)	48	86
	POST(EAP-Success)	38	76
	ACK	23	61
	**Total**	**311**	**577**
	PCI	16	54
	PAR	40	78
	PAN	40	78
	PARReq(Id)	48	86
	PANRep(Id)	60	98
PANA	PAR(EAP-PSK1)	56	94
	PAN(EAP-PSK2)	84	122
	PAR(EAP-PSK3)	84	122
	PAN(EAP-PSK4)	68	106
	PAR(EAP-Success)	88	126
	PAN	52	90
	**Total**	**636**	**1054**

## Abbreviations

The following abbreviations are used in this manuscript:

AAA	Authentication, Authorization, and Accounting
CoAP	Constrained Application Protocol
DTLS	Datagram Transport Layer Security
EAP	Extensible Authentication Protocol
eNodeB	NB-IoT Base Station
HTTP	Hypertext Transfer Protocol
I-D	Internet Draft
IoT	Internet of Things
LPWAN	Low Power Wide Area Networks
NB-IoT	Narrowband IoT
OMA	Open Mobile Alliance
PAA	PANA Agent
PANA	Protocol for Carrying Authentication for Network Access
PaC	PANA Client
RFC	Request for Comments
SO	Smart Object
TLS	Transport Layer Security
WSN	Wireless Sensor Networks

## References

[1] Gubbi J., Buyya R., Marusic S., Palaniswami M. (2013). Internet of Things (IoT): A vision, architectural elements, and future directions. Future Gener. Comput. Syst..

[2] Lichtblau K., Stich V., Bertenrath R., Blum M., Bleider M., Millack A., Schmitt K., Schmitz E., Schröter M. (2015). IMPULS-industrie 4.0-readiness. Impuls-Stiftung des VDMA, Aachen-Köln.

[3] Ratasuk R., Vejlgaard B., Mangalvedhe N., Ghosh A. NB-IoT system for M2M communication. Proceedings of the 2016 IEEE Wireless Communications and Networking Conference.

[4] Sornin N., Luis M., Eirich T., Kramp T., Hersent O. (2015). LoRaWAN specification. LoRa Alliance.

[5] Sigfox S., SIGFOX One Network A Billion Dreams (2016). M2M and IoT Redefined Through Cost Effective and Energy Optimized Connectivity. https://lafibre.info/images/3g/201302_sigfox_whitepaper.pdf.

[6] Sanchez-Iborra R., Cano M.D. (2016). State of the art in LP-WAN solutions for industrial IoT services. Sensors.

[7] Minaburo A., Gomez C., Toutain L., Paradells J., Crowcroft J. (2016). LPWAN Survey and GAP Analysis. https://datatracker.ietf.org/doc/html/draft-minaburo-lpwan-gap-analysis-02.

[8] 3GPP Security Architecture and Procedures for 5G System. http://www.3gpp.org/ftp//Specs/archive/33_series/33.501/33501-f50.zip.

[9] Bormann C., Ersue M., Keränen A. (2014). Terminology for Constrained-Node Networks.

[10] Garcia-Carrillo D., Marin-Lopez R., Kandasamy A., Pelov A. (2017). A CoAP-Based Network Access Authentication Service for Low-Power Wide Area Networks: LO-CoAP-EAP. Sensors.

[11] Gimenez O., Petrov I. (2019). Static Context Header Compression (SCHC) over LoRaWAN. https://datatracker.ietf.org/doc/html/draft-ietf-lpwan-schc-over-lorawan-04.

[12] Farrell S. (2018). Low-Power Wide Area Network (LPWAN) Overview. http://tools.ietf.org/rfc/rfc8376.txt.

[13] Garcia-Carrillo D., Lopez R., Kandasamy A., Pelov A. (2016). LoRaWAN Authentication in Diameter. https://datatracker.ietf.org/doc/html/draft-garcia-dime-diameter-lorawan-00.

[14] Garcia-Carrillo D., Lopez R., Kandasamy A., Pelov A. (2017). LoRaWAN Authentication in RADIUS. https://datatracker.ietf.org/doc/html/draft-garcia-radext-radius-lorawan-03.

[15] Open MobileAlliance (2013). Lightweight machine to machine requirements (White Paper). Candidate Version.

[16] Selander G., Mattsson J., Palombini F., Seitz L. (2019). Object Security for Constrained RESTful Environments (OSCORE). http://tools.ietf.org/rfc/rfc8613.txt.

[17] Zhang X., Kunz A., Schröder S. Overview of 5G security in 3GPP. Proceedings of the 2017 IEEE Conference on Standards for Communications and Networking (CSCN).

[18] Prasad A.R., Arumugam S., Sheeba B., Zugenmaier A. (2018). 3GPP 5G Security. J. ICT Stand..

[19] 3GPP (2019). 3rd Generation Partnership Project; Technical Specification Group Services and System Aspects; Release 16 Description; Summary of Rel-16 Work Items. http://ftp.3gpp.org//Specs/archive/21_series/21.916/21916-010.zip.

[20] Sanchez-Iborra R., Sánchez-Gómez J., Pérez S., Fernández P.J., Santa J., Hernández-Ramos J.L., Skarmeta A.F. (2018). Enhancing LoRaWAN Security through a Lightweight and Authenticated Key Management Approach. Sensors.

[21] Marin-Lopez R., Pereniguez-Garcia F., Gomez-skarmeta A.F., Ohba Y. (2012). Network access security for the internet: protocol for carrying authentication for network access. IEEE Commun. Mag..

[22] Sanchez P.M., Lopez R.M., Skarmeta A.F.G. (2013). PANATIKI: A Network Access Control Implementation Based on PANA for IoT Devices. Sensors.

[23] Österlind F. (2006). A Sensor Network Simulator for the Contiki OS.

[24] Garcia-Carrillo D. (2019). A CoaP-Based Bootstrapping Service for Large-Scale Internet-of-Things Networks. Ph.D. Thesis.

[25] de Laat C., Gross G., Gommans L., Vollbrecht J., Spence D. (2000). Generic AAA Architecture. https://rfc-editor.org/rfc/rfc2903.txt.

[26] Vollbrecht J., Carlson J., Blunk L., Aboba B. (2004). Extensible Authentication Protocol (EAP). https://rfc-editor.org/rfc/rfc3748.txt.

[27] Shelby Z., Hartke K., Bormann C. (2014). The Constrained Application Protocol (CoAP). https://rfc-editor.org/rfc/rfc7252.txt.

[28] Sarikaya B., Sethi M., Garcia-Carillo D. (2019). Secure IoT Bootstrapping: A Survey. https://datatracker.ietf.org/doc/html/draft-sarikaya-t2trg-sbootstrapping-07.

[29] Garcia-Morchon O., Kumar S., Sethi M. (2019). Internet of Things (IoT) Security: State of the Art and Challenges. https://rfc-editor.org/rfc/rfc8576.txt.

[30] Garcia-Carrillo D., Marin-Lopez R. (2016). Lightweight coap-based bootstrapping service for the internet of things. Sensors.

[31] Simon D., Eronen P. (2008). Extensible Authentication Protocol (EAP) Key Management Framework. http://www.3gpp.org/ftp/Specs/archive/45_series/45.820/45820-d10.zip.

[32] 3GPP (2015). Cellular System Support for Ultra-Low Complexity and Low throughput Internet of Things (CIoT). http://www.3gpp.org/ftp/Specs/archive/45_series/45.820/45820-d10.zip.

[33] Louis Columbus F. (2018). Roundup Of Internet Of Things Forecasts And Market Estimates. https://www.forbes.com/sites/louiscolumbus/2018/12/13/2018-roundup-of-internet-of-things-forecasts-and-market-estimates/.

[34] Tzounis A., Katsoulas N., Bartzanas T., Kittas C. (2017). Internet of Things in agriculture, recent advances and future challenges. Biosyst. Eng..

[35] WWAP (2015). The United Nations World Water Development Report 2015: Water for a Sustainable World.

[36] de Châtel F., Holst-Warhaft G., Steenhuis T.S. (2014). Water Scarcity, Security and Democracy: A Mediterranean Mosaic.

[37] NEEA (2015). Agricultural Irrigation Initiative: The Future of Agricultural Irrigation, Document Prepared by Marshall English.

[38] Stafford J.V. (2019). Precision Agriculture’19.

[39] (2019). European Cybersecurity Certification Framework. https://ec.europa.eu/digital-single-market/en/eu-cybersecurity-act.

[40] Xu L.D., He W., Li S. (2014). Internet of Things in Industries: A Survey. IEEE Trans. Ind. Inf..

[41] Dierks T., Rescorla E. (2008). The Transport Layer Security (TLS) Protocol Version 1.2. RFC 5246. https://tools.ietf.org/html/rfc5246.

[42] Wang Y.E., Lin X., Adhikary A., Grovlen A., Sui Y., Blankenship Y., Bergman J., Razaghi H.S. (2017). A Primer on 3GPP Narrowband Internet of Things. IEEE Commun. Mag..

[43] Andres-Maldonado P., Lauridsen M., Ameigeiras P., Lopez-Soler J.M. (2019). Analytical Modeling and Experimental Validation of NB-IoT Device Energy Consumption. IEEE Int. Things J..

[44] Lauridsen M., Krigslund R., Rohr M., Madueno G. An Empirical NB-IoT Power Consumption Model for Battery Lifetime Estimation. Proceedings of the 2018 IEEE 87th Vehicular Technology Conference (VTC Spring).

[45] Andres-Maldonado P., Ameigeiras P., Prados-Garzon J., Navarro-Ortiz J., Lopez-Soler J.M. (2019). An Analytical Performance Evaluation Framework for NB-IoT. IEEE Int. Things J..

[46] Mwakwata C.B., Malik H., Mahtab Alam M., Le Moullec Y., Parand S., Mumtaz S. (2019). Narrowband Internet of Things (NB-IoT): From Physical (PHY) and Media Access Control (MAC) Layers Perspectives. Sensors.

[47] Landström S., Bergström J., Westerberg E., Hammarwall D. (2016). NB-IOT: A sustainable technology for connecting billions of devices. Ericsson Rev..

[48] Feltrin L., Tsoukaneri G., Condoluci M., Buratti C., Mahmoodi T., Dohler M., Verdone R. (2019). Narrowband IoT: A Survey on Downlink and Uplink Perspectives. IEEE Wirel. Commun..

[49] 3GPP (2019). Evolved Universal Terrestrial Radio Access (E-UTRA); Medium Access Control (MAC) Protocol Specification. http://www.3gpp.org/ftp//Specs/archive/36_series/36.321/36321-f70.zip.

[50] 3GPP (2019). Evolved Universal Terrestrial Radio Access (E-UTRA); Radio Link Control (RLC) protocol specification. http://www.3gpp.org/ftp//Specs/archive/36_series/36.322/36322-f30.zip.

[51] 3GPP (2019). Evolved Universal Terrestrial Radio Access (E-UTRA); Packet Data Convergence Protocol (PDCP) specification. http://www.3gpp.org/ftp//Specs/archive/36_series/36.323/36323-f40.zip.

